# Association of dietary intake with micronutrient deficiency in Indian school children: a cross-sectional study

**DOI:** 10.1017/jns.2023.83

**Published:** 2023-10-02

**Authors:** Shally Awasthi, Divas Kumar, Swati Dixit, Abbas Ali Mahdi, Barkha Gupta, Girdhar G. Agarwal, Anuj Kumar Pandey, Avivar Awasthi, Somashekar A. R., Mushtaq A. Bhat, Sonali Kar, B. N. Mahanta, Joseph L. Mathew, Suma Nair, C. M. Singh, Kuldeep Singh, Anish Thekkumkara Surendran

**Affiliations:** 1 Department of Pediatrics, King George's Medical University, Lucknow, Uttar Pradesh, India; 2 Department of Biochemistry, King George's Medical University, Lucknow, Uttar Pradesh, India; 3 Lead-Nutritional Claims & Medical Affairs (Global HFD), HUL R&D Centre, Gurgaon, India; 4 Department of Statistics, University of Lucknow, Lucknow, Uttar Pradesh, India; 5 Department of Endocrinology, Kasturba Medical College, Manipal, Karnataka, India; 6 Department of Pediatrics, M. S. Ramaiah Institute of Medical Sciences, Bangalore, Karnataka, India; 7 Department of Pediatrics, Sher-i-Kashmir Institute of Medical Sciences, Srinagar, Jammu & Kashmir, India; 8 Department of Community Medicine, Kalinga Institute of Medical Sciences, Bhubaneswar, Orissa, India; 9 Department of Medicine, Assam Medical College, Dibrugarh, Assam, India; 10 Department of Pediatric Medicine, Post Graduate Institute of Medical Sciences, Chandigarh, India; 11 Department of Community Medicine, Kasturba Medical College, Manipal, Karnataka, India; 12 Department of Community & Family Medicine, All India Institute of Medical Sciences, Patna, Bihar, India; 13 Department of Pediatrics, All India Institute of Medical Sciences, Jodhpur, Rajasthan, India; 14 Department of Community Medicine, Government Medical College, Thiruvananthapuram, Kerala, India

**Keywords:** Childhood and adolescence, Deficiencies, Dietary intake, India, Micronutrients, School age children

## Abstract

Adequate nutrition is necessary during childhood and early adolescence for adequate growth and development. Hence, the objective of the study was to assess the association between dietary intake and blood levels of minerals (calcium, iron, zinc, and selenium) and vitamins (folate, vitamin B12, vitamin A, and vitamin D) in urban school going children aged 6–16 years in India, in a multicentric cross-sectional study. Participants were enrolled from randomly selected schools in ten cities. Three-day food intake data was collected using a 24-h dietary recall method. The intake was dichotomised into adequate and inadequate. Blood samples were collected to assess levels of micronutrients. From April 2019 to February 2020, 2428 participants (50⋅2 % females) were recruited from 60 schools. Inadequate intake for calcium was in 93⋅4 % (246⋅5 ± 149⋅4 mg), iron 86⋅5 % (7⋅6 ± 3⋅0 mg), zinc 84⋅0 % (3⋅9 ± 2⋅4 mg), selenium 30⋅2 % (11⋅3 ± 9⋅7 mcg), folate 73⋅8 % (93⋅6 ± 55⋅4 mcg), vitamin B12 94⋅4 % (0⋅2 ± 0⋅4 mcg), vitamin A 96⋅0 % (101⋅7 ± 94⋅1 mcg), and vitamin D 100⋅0 % (0⋅4 ± 0⋅6 mcg). Controlling for sex and socioeconomic status, the odds of biochemical deficiency with inadequate intake for iron [AOR = 1⋅37 (95 % CI 1⋅07–1⋅76)], zinc [AOR = 5⋅14 (95 % CI 2⋅24–11⋅78)], selenium [AOR = 3⋅63 (95 % CI 2⋅70–4⋅89)], folate [AOR = 1⋅59 (95 % CI 1⋅25–2⋅03)], and vitamin B12 [AOR = 1⋅62 (95 %CI 1⋅07–2⋅45)]. Since there is a significant association between the inadequate intake and biochemical deficiencies of iron, zinc, selenium, folate, and vitamin B12, regular surveillance for adequacy of micronutrient intake must be undertaken to identify children at risk of deficiency, for timely intervention.

## Introduction

Malnutrition has been defined as undernutrition, micronutrient-related malnutrition, and overweight/obesity.^(1)^ Undernutrition includes wasting (low weight-for-height), stunting (low height-for-age), and underweight (low weight-for-age). Since there is rapid growth during childhood and early adolescence (6–16 years), therefore adequate nutrition is required.

Deficiency of two or more micronutrients is significantly associated with decreased cognitive performance.^(2)^ Review of the literature suggests that deficiencies of iron, folate, and vitamin B12 can lead to anaemia, which further leads to delayed cognitive and motor development, low intelligent quotient, difficulties in functioning of work, education and community engagement.^(3,4)^ Folate deficiency is also reported to be associated with loss of concentration.^(5)^ Zinc deficiency interferes with the development of brain and cognition.^(6)^ Vitamin A deficiency is the leading cause of blindness and increases the risk of death from severe infections in children.^(3)^ Calcium and vitamin D are essential for the growth and development of the skeletal system. Selenium status is found to be associated with the cure rate of COVID-19.^(7)^

Comprehensive National Nutrition Survey (CNNS) 2019 reported one fourth of children and adolescents were thin in India, of which 5⋅0–6⋅5 % were severely thin. It also reported that 3⋅7–4⋅8 % were overweight and 1⋅1–1⋅3 % were obese in this age group.^(8)^ Small- and large-scale studies conducted so far in Indian school age children and adolescents, report that micronutrient intake is lower than recommended dietary allowance^(9)^ and the burden of biochemical deficiency of micronutrients is large.^(10)^ There is a paucity of researches studying the relation of dietary intake of micronutrients and its corresponding biochemical deficiencies in urban Indian children and adolescents especially from India. The present study was undertaken to study the association between dietary intake and blood levels of minerals (calcium, iron, zinc, and selenium) and vitamins (folate, vitamin B12, vitamin A, and vitamin D) in urban school going children aged 6–16 years in India.

## Methodology

### Ethical approval

This study was conducted according to the guidelines laid down in the Declaration of Helsinki and all procedures involving human subjects/patients were approved by the Institutional Ethics Committees for MS Ramaiah Medical College and Hospital Bangalore (approval reference number (ARN): MSRMC/EC/AP-02/02-2019), Kalinga Institute of Medical Sciences Bhubaneswar (ARN: KIMS/KIIT/IEC/112/2016), PGIMER Chandigarh (ARN: PGI/IEC/2019/000152), Assam Medical College (ARN: AMC/EC/1430), All India Institute of Medical Sciences Jodhpur (ARN: AIIMS/IEC/2017/765), King Georges Medical University (ARN: 9334/Ethics/R.Cell-16), Kasturba Medical College (ARN: IEC:388/2019), All India Institute of Medical Sciences Patna (ARN: IEC/AIIMS/PAT/153/2017), Sher-i-Kashmir Institute of Medical Sciences (ARN: IEC/SKIMS Protocol # RP 175/2018), and Medical College Thiruvananthapuram (ARN: HEC.No.04/34/2019/MCT). Written informed consent was obtained from parents of all study participants. The study was registered prospectively with the Clinical Trial Registry of India (registration number CTRI/2019/02/017783) [Clinical Trials Registry – India (CTRI)].

### Study design and setting

This cross-sectional multicentric study was conducted in ten cities of India, namely, Bangalore, Bhubaneswar, Chandigarh, Dibrugarh, Jodhpur, Lucknow, Udupi (Manipal), Patna, Srinagar, and Thiruvananthapuram, centrally coordinated by King George's Medical University (KGMU), Lucknow. The detailed study protocol is published elsewhere.^(1)^

### Sample size computation

Assuming the prevalence of folate deficiency in India as 30⋅7 %,^(11)^ then with precision (*d*) of 2 % and level of significance (*α*) of 0⋅05, the estimated sample size^(12)^ was 2044 participants. After taking, the attrition rate of 10⋅0 %, the sample size inflated to 2400 participants. This sample size was equally divided among the ten cities. To have balance, the sample was equally distributed between the two sexes. Sample size was calculated using folate deficiency as this gave the maximum sample.

### Selection of participants

Participants were selected by using a two-stage sampling technique. In the first stage, schools were selected and in the second stage, participants were recruited from the selected schools. Each study site provided a list of government as well as private schools enrolling children, both girls and boys, between 6 and 16 years of age and located within the city's urban limits. From this list, six schools were selected using simple random sampling, having at least one to a maximum of three private schools. Principals of the recruited schools were met to obtain voluntary written informed consent for school's participation. Then, with the help of an identified coordinating school teacher, a sex-wise list of students between 6 and 11 (group 1) and 12 and 16 years (group 2) of age was prepared in alphabetic order and numbered serially. From each of these lists, fifteen apparently healthy students, residing within 5 km of the school were selected by random number draw. They were invited to participate in the study. The first ten students whose parents provided written informed consent for participation were included in the study. The rest were kept as reserve. Written assent was obtained from all children older than 8 years.

Weight was measured to the nearest 0⋅1 kg using a portable Seca 803 weighing scale (Seca, Hamburg, Deutschland) and height was measured to the nearest 0⋅1 cm using Seca 213 Mobile Stadiometer (Seca, Hamburg, Deutschland). Body mass index (BMI) was calculated using the standard equation:
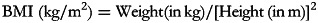


Those having BMI less than 12⋅5 were excluded from the study and their parents were informed and advised to seek medical consultation.

### Training of study team

To collect dietary data, a nutritionist was appointed at each site, who worked along with other team members. They had received a two-day training at KGMU, by SA and SD between March and July 2019, to record three 24-h recalls and enter this data into DIETSOFT software^(13)^ to obtain macro and micronutrient intake. Each site was given individual access to DIETSOFT. In addition, common training on study protocol, procedures, data collection methods, and instruments, was done by SA.

### Data collection

Participants and their primary caregivers were interviewed to record demographic, socioeconomic, and dietary intake data. Revised Kuppuswamy's socioeconomic scale was used to assess the socioeconomic status (SES) of participants^(14)^. Based on monthly family income, occupation, and education of head of family, this scale categorises SES as upper, upper middle, lower middle, upper lower, and lower classes.

#### 24-h dietary recall

The dietary habit was recorded as vegetarians, non-vegetarians, and eggetarians.^(15)^ Vegetarians were those who had never consumed animal products (except milk), eggetarians eat egg in addition to vegetarian food and non-vegetarians consume red meat/poultry/fish. Data on dietary intake of children was collected using a 24-h recall method for two non-consecutive weekdays and one Sunday, where there was no fasting or feasting. Any nutritional supplement taken by participants was also recorded. The quantity of intake was recorded, within 24 h, by interviewing participants along with the primary caregiver, preferably at their home. The cooked food items/recipe consumed, were recorded by its ingredients such as milk and sugar for tea, using standardised cups, glass, and spoons as an aid to help in recall of the amount used. For calculating the nutritive value of raw ingredients, the nutritive value of Indian foods was used.^(16)^ Nutritive value mentioned on package was used for packaged food items. Daily nutrient intake for all the nutrients was calculated using DIETSOFT software.^(13)^ The mean of 3-day intake of nutrients was calculated to obtain usual intake. This was compared with the estimated average requirement (EAR).^(17)^ Dietary intake was assessed in terms of intake adequacy (IA).^(17)^

The IA for a given nutrient was the ratio of a participant's intake to the current EAR by sex and age category. The IA was calculated for all the nutrients using the equation:

Intake adequacy = Participants nutrient intake of a day/EAR of the respective nutrient.

Participants were then categorised as having

- Adequate intake (IA ≥ 1⋅0) and
- inadequate intake (IA < 1⋅0).

### Blood sample collection

Blood sampling was done at the school by trained phlebotomists during school hours. Venous blood sample of 6 ml (4 ml in clot activator and 2 ml in EDTA vial) was collected using vacuum-tube systems, preferably from the cubital vein. All aseptic precautions were taken. Participants were kept under observation for 15 min after the sample collection.

During transportation of blood samples from school to study sites, temperature was maintained between 2 and 8°C, using pre-frozen gel packs in an ice box. Within 2 h, these samples were centrifuged at 1500 rpm for 10 min at 4°C to separate plasma, serum, and packed cells at the study site. Plasma and serum were stored below −20°C in trace element-free cryo tubes and packed cells between 2 and 8°C. Samples from study sites to KGMU were transported in two batches of 120 each, maintaining temperature below −20°C for plasma and serum and between 2 and 8°C for packed cells, by professional agencies having expertise in handling and shipment of biomedical samples. Samples were prevented from exposure to light during the whole process.

### Biochemical analysis

Blood samples were analysed to assess levels of calcium, iron, zinc, selenium, folate, vitamin B12, vitamin A, and vitamin D at the KGMU. The detailed methodology of biochemical analysis^(1)^ is published in earlier publications. Cut-off level to estimate micronutrient deficiencies is given in Table 1.

**Table 1.** Deficiency cut-off levels of micronutrients (in serum)

	Age group	Sex	Cut-off levels	Reference
Calcium	6–16 years	Male and Female	<10⋅0 mg/dl	(18)
Iron			<70⋅0 μg/dl	(19, 20)
Selenium			<5⋅5 μg/dl	(21)
Zinc	<10 years	Male and Female	<65⋅0 μg/dl	(22)
	≥10 years	Male	<70⋅0 μg/dl	
	≥10 years	Female	<66⋅0 μg/dl	
Vitamin A	6–16 years	Male and Female	<20⋅0 μg/dl	(23)
25-Hydroxy vitamin D			<12⋅0 ng/ml	(15)
Folate			<3⋅0 ng/ml	(24)
Vitamin B12			<203.0 pg/ml	(15)

### Quality assurance

Data from all the sites were sent monthly through email and the entire data was maintained and analysed at KGMU. Robust mechanisms were employed to maintain the quality of data collection. Data collection at sites was done under the direct supervision of the Site Investigator/Co-Investigator. KGMU monitored data quality by onsite monitoring to observe the data collection process and ascertain that this had been done as per laid standard operating procedures. Retraining was imparted where gaps were identified. Internal and external quality control of blood sample analysis was ensured by running controls along with the samples and participating in external quality assurance programmes.

### Statistical analysis

Double data entry was done in MS Excel. Data were matched electronically and discrepancies were rectified by referring to the source documents. Overall descriptive statistics of study participants such as sociodemographic, anthropometric indicators, and dietary habits were calculated and compared between sexes. SES was dichotomised into upper or upper middle or lower middle and upper lower or lower. The proportion of participants with deficiency of various micronutrients in the blood was calculated. Categorical variables were given in number and percentage. Mean, median, and interquartile range (IQR) were used for the continuous variable. Based on the EAR, the proportion of participants having adequate and inadequate dietary intake of micronutrients was calculated. Statistical analysis was performed using SPSS statistical software version 24.^(25)^

The student's *t* and Mann–Whitney *U* tests were used for the comparison of micronutrient intake between sex, age, and SES. Effect size with 95 % confidence interval (CI) was calculated to see the average difference between the two groups. We used analysis of variance (ANOVA) and Kruskal–Wallis test to compare the intake of micronutrients among categories of anthropometric indicators. The proportion of inadequate intake was compared to see the differences between sex, age, anthropometric indicators, and SES using the Chi-square test.

For univariate and multivariate logistic regression models, we have used only those cases where data of both dietary intake and level of micronutrient was available. The models were used to assess the association of inadequate intake with biochemical deficiency of micronutrients. Crude odds ratio (COR) and adjusted odds ratio (AOR) with a 95 % CI were calculated for micronutrient deficiency in blood adjusted for sex and SES. Two-tailed tests were used to test the hypothesis and a *P*-value < 0⋅05 was taken as statistically significant.

## Results

Data were collected from April 2019 to February 2020. There was almost equal sex distribution among 2428 participants enrolled. Table 2 shows the comparison of sociodemographic, anthropometric, and dietary habits of study participants by sex.

**Table 2.** Distribution of sociodemographic, anthropometric, and dietary habits of study participants by sex

	Overall (*N* 2428) *n*, %	Boys (*n* 1210)	Girls (*n* 1218)
Age (mean ± sd) in years	11⋅8 ± 2⋅4	11⋅8 ± 2⋅4	11⋅7 ± 2⋅4
School type (row %)			
Government	1524, 62⋅8	760, 49⋅9	764, 50⋅1
Private	904, 37⋅3	450, 49⋅8	454, 50⋅2
Type of family (row %)			
Joint	743/2425, 30⋅6	358/743, 48⋅2	385/743, 51⋅8
Nuclear	1682/2425, 69⋅4	849/1682, 50⋅5	833/1682, 49⋅5
Primary caregiver (row %)			
Mother	2056, 84⋅7	1020, 49⋅6	1036, 50⋅4
Father	151, 6⋅2	80, 53⋅0	71, 47⋅0
Others	221, 9⋅1	110, 49⋅8	111, 50⋅2
Socioeconomic class (Revised Kuppuswamy's socioeconomic scale) (row %)			
Upper or upper middle or lower middle	1403/2425, 57⋅9	717/1403, 51⋅1	686/1403, 48⋅9
Upper lower or lower	1022/2425, 42⋅1	490/1022, 48⋅0	532/1022, 52⋅0
Anthropometric Indicators (column %)			
Height for age			
Stunted (HAZ < –2 sd)	362, 14⋅9	169, 14⋅0	193, 15⋅8
Anthropometric indicator (column %)			
Severe thinness or thinness	359, 14⋅8	196, 16⋅2	163, 13⋅4
Normal	1682, 69⋅3	829, 68⋅5	853, 70⋅0
Overweight	263, 10⋅8	124, 10⋅2	139, 11⋅4
Obese or severe obese	124, 5⋅1	61, 5⋅0	63, 5⋅2
Dietary habit (column %)			
Vegetarian	376, 15⋅5	195, 16⋅1	181, 14⋅9
Non-vegetarian	1922, 79⋅2	952, 78⋅7	970, 79⋅6
Eggetarian	130, 5⋅3	63, 5⋅2	67, 5⋅5

In blood, iron deficiency was found in 49⋅4 % (1119/2265) participants, calcium deficiency in 20⋅6 % (468/2267), selenium in 10⋅4 % (208/2003), and zinc in 6⋅8 % (138/2026). Vitamin D was found to be deficient in 39⋅7 % (900/2268), Vitamin B12 in 33⋅4 % (759/2275), and folate in 22⋅2 % (505/2276) participants. Vitamin A deficiency or marginal deficiency was found in 7⋅7 % (174/2250).

**Fig. 1.**
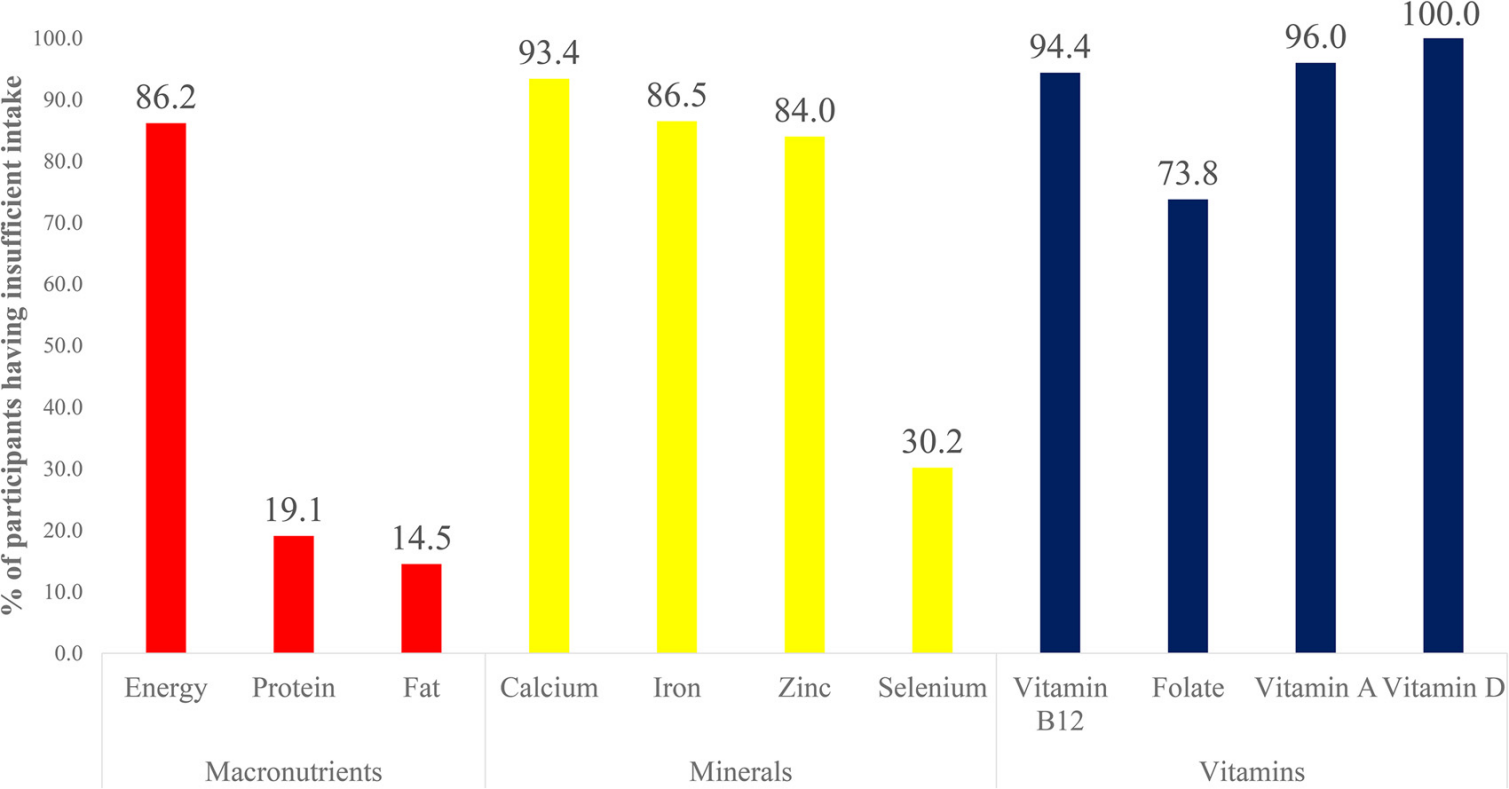
Distribution of participants on basis of inadequate intake of macro and micronutrients.

The distribution of participants on the basis of dietary inadequacy of various macro and micronutrients is shown in Fig. 1. A higher proportion of participants had inadequate dietary intake of minerals and vitamins except selenium.

The mean distribution of micronutrient intake by sex, age, and anthropometric indicators is shown in Table 3. There was a significantly higher mean intake of micronutrient in males as compared to females with an estimated effect size of 0⋅15 (95 % CI 0⋅07–0⋅23) for calcium, 0⋅26 (95 % CI 0⋅18–0⋅34) for iron, 0⋅26 (95 % CI 0⋅18–0⋅34) for zinc, 0⋅24 (95 % CI 0⋅16–0⋅32) for selenium, 0⋅12 (95 % CI 0⋅04–0⋅19) for vitamin A, and significantly lower mean intake of micronutrient in age group 6–11 years as compared to 12–16 years with estimated effect size of −0⋅21 (95 % CI −0⋅29 to −0⋅13) for iron, −0⋅20 (95 % CI −0⋅28 to −0⋅13) for zinc, −0⋅17 (95 % CI −0⋅24 to −0⋅86) for selenium, and −0⋅13 (−0⋅21 to −0⋅05) for folate. A significantly higher proportion of severely thin or thin participants had an inadequate intake of calcium and folate. Inadequate intake of selenium was a significantly higher in the overweight or obese or severely obese participants. Comparing mean intake of micronutrients among the category of SES, it was found that there was a significantly higher mean intake in upper, upper middle, or lower middle class for calcium and lower mean intake for zinc, selenium, and vitamin D. There was a significantly higher proportion of inadequate intake in upper or upper middle or lower middle vitamin for selenium and lower proportion of calcium, as shown in Table 4.

**Table 3.** Intake of micronutrients by sex, age, and anthropometric indicator

Intake of micronutrients (*n* 2428)	Sex			Age group (in years)			Anthropometric indicator			
	Male	Female	*P*-value	6–11	12–16	*P*-value	Normal	Severe thinness or thinness	Overweight or obese or severely obese	*P*-value
Calcium (in mg)										
Mean	300⋅2	268⋅5	<0⋅001	281⋅1	287⋅6	0⋅46	278⋅6	248⋅8	342⋅3	<0⋅001
Median, IQR	248⋅9, 255⋅5	225⋅9, 218⋅3	0⋅005	234⋅6, 233⋅4	241⋅9, 239⋅8	0⋅36	236⋅6, 227⋅9	211⋅5, 188⋅7	301⋅1, 326⋅5	<0⋅001
Inadequate intake *n*, %^a^	1117, 92⋅3	1151, 94⋅5	0⋅03	1116, 90⋅4	1152, 96⋅6	<0⋅001	1583, 94⋅1	347, 96⋅7	338, 87⋅3	<0⋅001
Iron (in mg)										
Mean	9⋅2	8⋅1	<0⋅001	8⋅2	9⋅1	<0⋅001	8⋅7	8⋅2	8⋅6	0⋅14
Median, IQR	8⋅5, 5⋅7	7⋅6, 4⋅7	<0⋅001	7⋅7, 4⋅8	8⋅5, 5⋅7	<0⋅001	8⋅1, 5⋅2	7⋅5, 5⋅0	8⋅2, 5⋅2	0⋅07
Inadequate intake *n*, %^a^	956, 79⋅0	1144, 93⋅9	<0⋅001	1026, 83⋅1	1074, 90⋅0	<0⋅001	1451, 86⋅3	314, 87⋅5	335, 86⋅6	0⋅83
Zinc (in mg)										
Mean	4⋅8	4⋅1	<0⋅001	4⋅2	4⋅7	<0⋅001	4⋅5	4⋅7	4⋅0	<0⋅001
Median, IQR	4⋅7, 3⋅7	4⋅1, 3⋅1	<0⋅001	4⋅1, 3⋅0	4⋅6, 3⋅8	<0⋅001	4⋅4, 3⋅2	4⋅6, 3⋅1	3⋅5, 4⋅6	<0⋅001
Inadequate intake *n*, %^a^	972, 80⋅3	1067, 87⋅6	<0⋅001	925, 74⋅9	1114, 93⋅4	<0⋅001	1409, 83⋅8	309, 86⋅1	321, 82⋅9	0⋅47
Selenium (in mcg)										
Mean	53⋅5	44⋅5	<0⋅001	45⋅9	52⋅2	<0⋅001	49⋅5	51⋅9	44⋅2	0⋅01
Median, IQR	46⋅7, 52⋅7	39⋅8, 43⋅8	<0⋅001	40⋅9, 44⋅2	46⋅1, 53⋅3	0⋅004	43⋅7, 47⋅5	45⋅1, 44⋅6	38⋅2, 55⋅2	0⋅003
Inadequate intake *n*, %^a^	350, 47⋅7	383, 52⋅3	0⋅18	382, 52⋅1	351, 47⋅9	0⋅42	497, 29⋅5	93, 25⋅9	143, 37⋅0	0⋅003
Vitamin A (in mcg)										
Mean	124⋅8	110⋅0	0⋅005	118⋅4	116⋅4	0⋅71	116⋅4	114⋅7	124⋅3	0⋅50
Median, IQR	91⋅6, 152⋅8	78⋅3, 139⋅9	0⋅01	87⋅3, 143⋅0	83⋅7, 148⋅7	0⋅53	84⋅0, 147⋅8	78⋅1, 154⋅0	93⋅8, 138⋅2	0⋅77
Inadequate intake *n*, %^a^	1144, 94⋅5	1187, 97⋅5	<0⋅001	1166, 94⋅4	1165, 97⋅7	<0⋅001	1618, 96⋅2	346, 96⋅4	367, 94⋅8	0⋅43
Folate (in mcg)										
Mean	165⋅5	151⋅1	0⋅07	146⋅4	170⋅6	0⋅002	159⋅9	160⋅1	149⋅8	0⋅64
Median, IQR	128⋅7, 122⋅0	114⋅5, 108⋅1	<0⋅001	113⋅2, 106⋅3	130⋅2, 129⋅2	0⋅001	122⋅2, 112⋅1	120⋅9, 103⋅2	116⋅4, 147⋅0	0⋅42
Inadequate intake *n*, %^a^	869, 71⋅8	924, 75⋅9	0⋅02	867, 70⋅2	926, 77⋅6	<0⋅001	1245, 74⋅0	281, 78⋅3	267, 69⋅0	0⋅02
Vitamin B12 (in mcg)										
Mean	0⋅5	0⋅5	0⋅90	0⋅5	0⋅5	0⋅21	0⋅5	0⋅4	0⋅7	0⋅05
Median, IQR	0⋅04, 0⋅3	0⋅04, 0⋅3	0⋅72	0⋅05, 0⋅3	0⋅04, 0⋅3	0⋅20	0⋅0, 0⋅3	0⋅03, 0⋅2	0⋅2, 0⋅6	<0⋅001
Inadequate intake *n*, %^a^	1144, 94⋅5	1149, 94⋅3	0⋅82	1177, 95⋅3	1116, 93⋅5	0⋅06	1590, 94⋅5	341, 95⋅0	362, 93⋅5	0⋅66
Vitamin D (in mcg)										
Mean	0⋅4	0⋅3	0⋅17	0⋅4	0⋅3	0⋅44	0⋅4	0⋅5	0⋅3	<0⋅001
Median, IQR	0⋅0, 0⋅6	0⋅1, 0⋅5	0⋅95	0⋅0, 0⋅6	0⋅1, 0⋅5	0⋅96	0⋅1, 0⋅5	0⋅2, 0⋅7	0⋅0, 0⋅3	<0⋅001
Inadequate intake *n*, %^a^	1209, 49⋅8	1218, 50⋅2	0⋅32	1235, 50⋅9	1192, 49⋅1	0⋅31	1682, 100⋅0	358, 99⋅7	387, 100⋅0	0⋅06

a Percentage shown for inadequate intake is column percent.

**Table 4.** Intake of micronutrients by socioeconomic status

Intake of micronutrients (*n* 2428)	Socioeconomic status		
	Upper, upper middle, or lower middle	Upper lower or lower	*P*-value
Calcium (in mg)			
Mean	311⋅9	246⋅9	<0⋅001
Median, IQR	263⋅9, 279⋅5	214⋅5, 185⋅7	<0⋅001
Inadequate intake *n*, %^a^	1279, 91⋅2	986, 96⋅5	<0⋅001
Iron (in mg)			
Mean	8⋅7	8⋅5	0⋅26
Median, IQR	8⋅3, 5⋅3	7⋅7, 5⋅0	0⋅09
Inadequate intake *n*, %^a^	1200, 85⋅5	897, 87⋅8	0⋅11
Zinc (in mg)			
Mean	4⋅3	4⋅7	<0⋅001
Median, IQR	4⋅2, 4⋅0	4⋅5, 2⋅9	<0⋅001
Inadequate intake *n*, %^a^	1182, 84⋅2	854, 83⋅6	0⋅65
Selenium (in mcg)			
Mean	47⋅7	50⋅9	0⋅04
Median, IQR	42⋅4, 53⋅6	43⋅8, 42⋅0	0⋅01
Inadequate intake *n*, %^a^	474, 64⋅9	256, 35⋅1	<0⋅001
Vitamin A (in mcg)			
Mean	116⋅5	118⋅8	0⋅66
Median, IQR	86⋅6, 144⋅7	84⋅4, 147⋅1	0⋅39
Inadequate intake *n*, %^a^	1345, 95⋅9	983, 96⋅2	0⋅69
Folate (in mcg)			
Mean	156⋅3	161⋅1	0⋅54
Median, IQR	114⋅2, 126⋅8	128⋅1, 105⋅0	<0⋅001
Inadequate intake *n*, %^a^	1042, 74⋅3	748, 73⋅2	0⋅55
Vitamin B12 (in mcg)			
Mean	0⋅5	0⋅4	0⋅07
Median, IQR	0⋅1, 0⋅4	0⋅02, 0⋅2	0⋅72
Inadequate intake *n*, %^a^	1316, 93⋅8	975, 95⋅4	0⋅09
Vitamin D (in mcg)			
Mean	0⋅3	0⋅4	<0⋅001
Median, IQR	0⋅0, 0⋅4	0⋅2, 0⋅7	<0⋅001
Inadequate intake *n*, %^a^	1402, 57⋅8	1022, 42⋅2	0⋅39

a Percentage shown for inadequate intake is column percent.

Inadequate intakes of micronutrients had a significant association with the crude odds of biochemical deficiency. The association of inadequate intake with biochemical deficiency of micronutrients adjusted for sex and SES is shown in Table 5. The AOR of inadequate intake of iron, zinc, selenium, folate, vitamin B12, and vitamin A showed higher odds of biochemical deficiency when adjusting for sex and SES.

**Table 5.** Association of inadequate intake with biochemical deficiency of micronutrients adjusted for sex and socioeconomic status

Nutritional Adequacy *n* (%)	Biochemical level of micronutrient			
	Deficiency	No deficiency	Crude OR (95 % CI)	Adjusted OR (95 % CI)
Calcium				
Adequate	92 (6⋅8)	59 (6⋅5)	–	–
Inadequate	1266 (93⋅2)	850 (93⋅5)	0⋅96 (0⋅68–1⋅34) *P* = 0⋅79	0⋅89 (0⋅63–1⋅26) *P* = 0⋅51
Iron				
Adequate	123 (11⋅0)	185 (16⋅1)	–	–
Inadequate	996 (89⋅0)	961 (83⋅9)	1⋅56 (1⋅22–1⋅99) *P* < 0⋅001	1⋅37 (1⋅07–1⋅76) *P* = 0⋅01
Zinc				
Adequate	6 (4⋅3)	335 (17⋅7)	–	–
Inadequate	132 (95⋅7)	1553 (82⋅3)	4⋅75 (2⋅08–10⋅85) *P* < 0⋅001	5⋅14 (2⋅24–11⋅78) *P* < 0⋅001
Selenium				
Adequate	84 (40⋅4)	1292 (72⋅0)	–	–
Inadequate	124 (59⋅6)	503 (28⋅0)	3⋅79 (2⋅82–5⋅10) *P* < 0⋅001	3⋅63 (2⋅70–4⋅89) *P* < 0⋅001
Folate				
Adequate	100 (19⋅8)	502 (28⋅3)	–	–
Inadequate	405 (80⋅2)	1269 (71⋅7)	1⋅60 (1⋅26–2⋅04) *P* < 0⋅001	1⋅59 (1⋅25–2⋅03) *P* < 0⋅001
Vitamin B12				
Adequate	31 (4⋅1)	101 (6⋅7)	–	–
Inadequate	728 (95⋅9)	1415 (93⋅3)	1⋅68 (1⋅11–2⋅53) *P* = 0⋅01	1⋅62 (1⋅07–2⋅45) *P* = 0⋅02
Vitamin A^a^				
Adequate	18 (10⋅3)	72 (3⋅5)	–	–
Inadequate	156 (89⋅7)	2004 (96⋅5)	0⋅31 (0⋅18–0⋅54) *P* < 0⋅001	0⋅31 (0⋅18–0⋅54) *P* < 0⋅001

OR, odds ratio; CI, confidence interval.

a Deficiency or marginal deficiency.

Adequate is taken as reference.

The sensitivity analysis of mean dietary intake of micronutrients on weekdays and weekend shows no significant difference.

## Discussion

This cross-sectional study was conducted to assess the association between the dietary intake and blood levels of minerals (calcium, iron, zinc, and selenium) and vitamins (folate, vitamin B12, vitamin A, and vitamin D) in urban school going children aged 6–16 years in ten cities of India. We found a significant positive association between dietary intake and blood concentration of calcium, iron, zinc, selenium, vitamin B12, and folate. Every three out of four participants had an inadequate intake of calcium, iron, zinc, and vitamin B12 in their diet. One-third of participants reported inadequate intake of selenium and folate. Almost all the participants were having inadequate intake of vitamin A and D. Dietary intake of females is more inadequate for calcium, iron, zinc, and folate as compared to males. Similarly, the diet of older age group participants was inadequate in calcium, iron, and zinc. In blood, almost half of the participants were deficient in iron, one-third in vitamin D and B12, and one-fifth in calcium and folate.^(10)^ Selenium, zinc, and vitamin A deficiencies were found in ≤10 %.^(10)^

The dietary intake was recorded for two weekdays and a weekend (Sunday), excluding fasting and feasting days, using a robust 24-h dietary recall method. To generalise the regional variability and diversity of diets, nutritional values were calculated using DIETSOFT software,^(13)^ which has been extensively used in Indian studies.^(26,27)^ DIETSOFT software takes the Indian dietetic scenario into account. DIETSOFT contains 1420 commonly consumed Indian food items based on the India Food Composition Tables (IFCT)^(28)^ and the National Institute of Nutrition (NIN) Nutritive Value of Indian Foods list.^(16)^ Several other food items relevant to local diets which were not available in IFCT and NIN were added to the software by the nutritionists. Estimation of micronutrients in blood was done using standardised methods, which are published in detail elsewhere.^(1)^

The children in the study had suboptimal diets and low adequate intake of almost all micronutrients. This is in line with a study conducted in South India, that reported an inadequate intake of iron, vitamin B12, and folate in diet of peri-urban school children.^(29)^ Another study from Manipur, an eastern state of India, reported children and adolescents have low dietary inadequacy of calcium and vitamin A.^(30)^ The National Nutrition Monitoring Bureau (2011–12) reported that adequacy of calcium, iron, zinc, folate, vitamin A, and vitamin B12 intake was very poor in adolescent girls from ten states of India.^(31)^ In a literature review on food and nutrient intake, the authors concluded that food items rich in micronutrients are generally consumed less frequently in India.^(9)^ More than half of the participants (53⋅1 %) in the current study had inadequate energy intake. Studies from Bhopal (Madhya Pradesh, India) among school going children^(32)^ and Delhi among adolescent girls^(33)^ reported deficient energy intake. We found that out of every five participants, four had adequate intake of protein and fat. A study among adolescents in rural India reported similar findings.^(34)^

We observed a significant positive association between dietary intake and blood concentration of calcium, iron, zinc, selenium, vitamin B12, and folate. A similar positive correlation was found in adults from the Netherlands between the dietary intake and blood concentrations of folate and vitamin B12.^(35)^ Another study among premenopausal women in Australia reported the positive association between intake and blood levels of iron and zinc.^(36)^ However, another study among Australian children and adolescents found that dietary zinc intake was not correlated with serum zinc concentration.^(37)^ A study from Austria found a significant positive association of selenium intake with blood level among patients attending thyroid clinic.^(38)^ Similar association was reported by a study from New Zealand among children aged 5–14 years.^(39)^ Association between dietary intake and blood levels of calcium was reported among pregnant women from Ethiopia.^(40)^ However, we did not come across any study from India, which assess the association between dietary intake and blood concentration of micronutrients in children and adolescents.

The current study shows that inadequate vitamin A intake is 96⋅2 % while deficiency or marginal deficiency in blood was only 7⋅7 %. We observed an inverse association between its dietary intake and blood level [AOR 0⋅31 (95 % CI 0⋅18–0⋅54)]. At times of inadequate dietary intake, levels of vitamin A in blood are maintained by mobilising it from liver. Low circulating levels of vitamin A are truly the reflection of disturbed vitamin A metabolism rather than current vitamin A deficiency.^(41)^

Since there are no good dietary sources of vitamin D, so all the participants in the present study had inadequate intake of vitamin D in their diet, however those deficient in blood were 39⋅7 %. We could not assess the association of biochemical level and nutritional adequacy of vitamin D. The vitamin D should be supplemented in food as per EAR.

Some of the studies have reported that peer influences, availability and access to ready to eat energy-dense food, nutrient poor convenience foods at homes, schools, and urban neighbourhoods and eye-catching repeated commercials on fast foods in mass media, perhaps are the undesirable influencers on food habits of children and adolescents in urban India.^(22)^ The above factors were not studied but likely to exist and may have contributed to children developing unhealthy food habits leading to micronutrient deficiencies. Further studies are needed in this area.

The current multicentric study had adequate sample size and was conducted to study the association of various crucial micronutrients in blood with their intake. This is probably the first study to assess the nutritional intake adequacy on basis of EAR and to assess the association of dietary intake and biochemical levels of micronutrients in the Indian population. However, there was one-time measurement of blood micronutrient levels, dietary intake and longitudinal follow-up was not done. Another limitation was that factors affecting the bioavailability of nutrients were not considered. Possibility of recall bias during dietary data collection cannot be completely ruled out.

## Conclusion

In India, the micronutrient deficiency continues to persist among school children and adolescents. Since there is a significant association between dietary intake and micronutrient deficiencies in blood, hence periodic surveillance through dietary recall surveys could be undertaken to identify children at risk of micronutrient deficiency. Dietary recall survey is less expensive, easy to conduct, and non-invasive.
